# Assessing cellular internalization and endosomal escape abilities of novel BUFII-Graphene oxide nanobioconjugates

**DOI:** 10.3389/fchem.2022.974218

**Published:** 2022-09-15

**Authors:** Julian Daniel Torres-Vanegas, Javier Cifuentes, Paola Ruiz Puentes, Valentina Quezada, Andres J. Garcia-Brand, Juan C. Cruz, Luis H. Reyes

**Affiliations:** ^1^ Department of Chemical and Food Engineering, Grupo de Diseño de Productos y Procesos (GDPP), Universidad de Los Andes, Bogotá, Colombia; ^2^ Department of Biomedical Engineering, Universidad de Los Andes, Bogotá, Colombia

**Keywords:** nanobioconjugates, graphene oxide, endosomal escape, cell internalization, buforin II

## Abstract

Cell-penetrating agents based on functionalized nanoplatforms have emerged as a promising approach for developing more efficient and multifunctional delivery vehicles for treating various complex diseases that require reaching different intracellular compartments. Our previous work has shown that achieving full cellular coverage and high endosomal escape rates is possible by interfacing magnetite nanoparticles with potent translocating peptides such as Buforin II (BUF-II). In this work, we extended such an approach to two graphene oxide (GO)-based nanoplatforms functionalized with different surface chemistries to which the peptide molecules were successfully conjugated. The developed nanobioconjugates were characterized via spectroscopic (FTIR, Raman), thermogravimetric, and microscopic (SEM, TEM, and AFM) techniques. Moreover, biocompatibility was assessed via standardized hemocompatibility and cytotoxicity assays in two cell lines. Finally, cell internalization and coverage and endosomal escape abilities were estimated with the aid of confocal microscopy analysis of colocalization of the nanobioconjugates with Lysotracker Green^®^. Our findings showed coverage values that approached 100% for both cell lines, high biocompatibility, and endosomal escape levels ranging from 30 to 45% and 12–24% for Vero and THP-1 cell lines. This work provides the first routes toward developing the next-generation, carbon-based, cell-penetrating nanovehicles to deliver therapeutic agents. Further studies will be focused on elucidating the intracellular trafficking pathways of the nanobioconjugates to reach different cellular compartments.

## 1 Introduction

The quality of life of people worldwide has been affected by several diseases, such as cancer, neurodegenerative disorders, orphan diseases, and bacterial and fungal infections, among many others (The top 10 causes of death, 2021). To treat these conditions, several therapeutic compounds have been considered, such as small molecules (e.g., chemotherapeutics, antidepressants, anti-inflammatory, and antioxidant compounds) (Dean et al., 2005; Deshpande et al., 2013; Rodzinski et al., 2016; Ezrahi et al., 2019; Pereira et al., 2021; Yang and Xie, 2021), biologicals (Adki and Kulkarni, 2020; Iglesias et al., 2020; Nguyen et al., 2020; Wang et al., 2020), and antifungal and antibacterial agents (Mudavath et al., 2014; Lazaridou et al., 2021; Hsiung et al., 2022). However, the delivery of these therapeutics to specific intracellular targets usually occurs with very low efficiencies, and only small fractions of the molecules can reach the sites of action. Also, the potential side effects of these treatments are generally unknown. These major issues have been the focus of a large body of research in pharmacology for the past three decades.

The fact that such therapeutics exhibit low efficiencies is because they must pass through several biological barriers, including physiological systems (e.g., their clearance by the liver or kidney), extracellular defense mechanisms (e.g., their uptake by the macrophage system (MPS)), and intracellular barriers such as their entrapment by endosomes (Homayun et al., 2019; Torres-Vanegas et al., 2021). In this regard, endosomal compartments are vesicles capable of capturing the nanostructured systems and causing their enzymatic degradation, which decreases their functional therapeutic effect in the target tissue (Hou et al., 2015; Ahmad et al., 2019; Smith et al., 2019). Given the need to avoid endosomal vesicles’ entrapment of the nanovehicles, extensive research over the past two decades has focused on engineering strategies to address this issue.

Nanomaterial-based delivery systems have gained considerable attention to efficiently deliver cargoes in a targeted manner and surpass the biological barriers upon administration (Ravichandran, 2009; Chou et al., 2011; Chao et al., 2021; Nguyen et al., 2021; Sahu et al., 2021; Younas et al., 2021). These nanosystems comprise two main components: a nanomaterial support or carrier and a functionalizing biomolecule. Among an ample diversity of nanomaterials used for the development of efficient delivery systems, graphene oxide (GO) has emerged as a promising alternative (Yang K. et al., 2012; Chimene et al., 2015; Yin et al., 2017; Hasan et al., 2019; Sandoval et al., 2019). Besides its low cost and scalable production, recent manufacturing approaches have provided routes for controlled thickness, maintaining a high surface area, and incorporating highly reactive chemical groups such as hydroxyls, epoxies, and carbonyls (de Melo-Diogo et al., 2018; Wei et al., 2021; Ajala et al., 2022). These groups make GO an ideal nanoplatform for functionalization with different biological and chemical molecules, improving its colloidal stability, biocompatibility, and cellular uptake.

Among the different strategies to functionalize the nanomaterials with bioactive agents, cell-penetrating peptides have become attractive to improve cell internalization and endosomal escape for these delivery nanoplatforms mainly due to their high efficiencies. Previously, we synthesized magnetite nanoparticles and modified their surface with the cell-penetrating and antimicrobial peptide Buforin II (BUF-II) (Cuellar et al., 2018; Perez et al., 2019; Ramírez-Acosta et al., 2020). This peptide has shown a potent ability to translocate the cell membrane (Muñoz-Camargo et al., 2018) and, when immobilized on magnetite nanoparticles, showed a solid ability to escape from endosomes upon internalization in several cell lines, including Vero, THP-1, HaCaT, HFF, and neuroblastoma cells (Cuellar et al., 2018; Perez et al., 2019; Ramírez-Acosta et al., 2020). Regarding the need to improve the efficiency of nanomaterial-based delivery systems and our recent advances concerning the ability of endosomal escape, this study extends the use of cell-penetrating peptides towards the functionalization of GO to develop effective and biocompatible nanoengineered vehicles.

In particular, we developed a methodology to synthesize and functionalize GO with reducible or polymeric molecules and the translocating peptide BUF-II. The resulting nanobioconjugates were characterized through spectroscopic, thermogravimetric, and microscopic techniques. Moreover, their biocompatibility, cell penetration, and endosomal escape abilities were evaluated in Vero and THP-1 cells. Our findings contribute to determining the impact of the functionalization strategy on the cell internalization and endosomal escape processes. In addition, this study provides valuable insights into a novel graphene-based nanoplatform to design more efficient nanovehicles rationally. Further studies will focus on elucidating the possible pathways involved in these penetration and intracellular trafficking processes.

## 2 Materials and methods

### 2.1 Reagents and cell cultures

Graphite powder was purchased from Graphene Supermarket (Ronkonkoma, NY, United States). Sulfuric acid (H_2_SO_4_), phosphoric acid (H_3_PO_4_), potassium permanganate (K_2_MnO_4_), hydrochloric acid (HCl), and hydrogen peroxide were purchased from PanReac AppliChem (Chicago, IL, United States). 3-[(2-amino ethyl) dithio] propionic acid·HCl (AEDP), NH_2_-PEG_12_-Propionic acid (PEG), N-[3-(dimethylamino)-propyl]-N′-ethyl carbodiimide hydrochloride (EDC), N-hydroxy-sulfosuccinimide (NHS), dimethyl-formamide (DMF), phosphate-buffered saline (PBS), 3-(4,5-dimethyl-2-thiazolyl)-2,5-diphenyl-2H-tetrazolium bromide (MTT), Rhodamine B, Propidium iodide, and Triton X-100 were purchased from Sigma-Aldrich (St. Louis, MO, United States). Buforin II (BUF-II: TRSSRAGLQFPVGRVHRLLRK) was synthesized by GL Biochem (Shanghai, China). High-glucose Dulbecco’s modified Eagle medium (DMEM) was purchased from Gibco (Amarillo, TX, United States). Fetal bovine serum (FBS) was purchased from BioWest (Riverside, MO, United States). Lysotracker Green DND-26 and Hoechst 33,342 were purchased from Thermo Fisher (Waltham, MA, United States). Vero cells (ATCC^®^ CCL-81) and THP-1 cells (ATCC^®^ TIB-202) were used for the cytotoxicity and cell internalization assays.

### 2.2 Synthesis of GO

**FIGURE 1:**
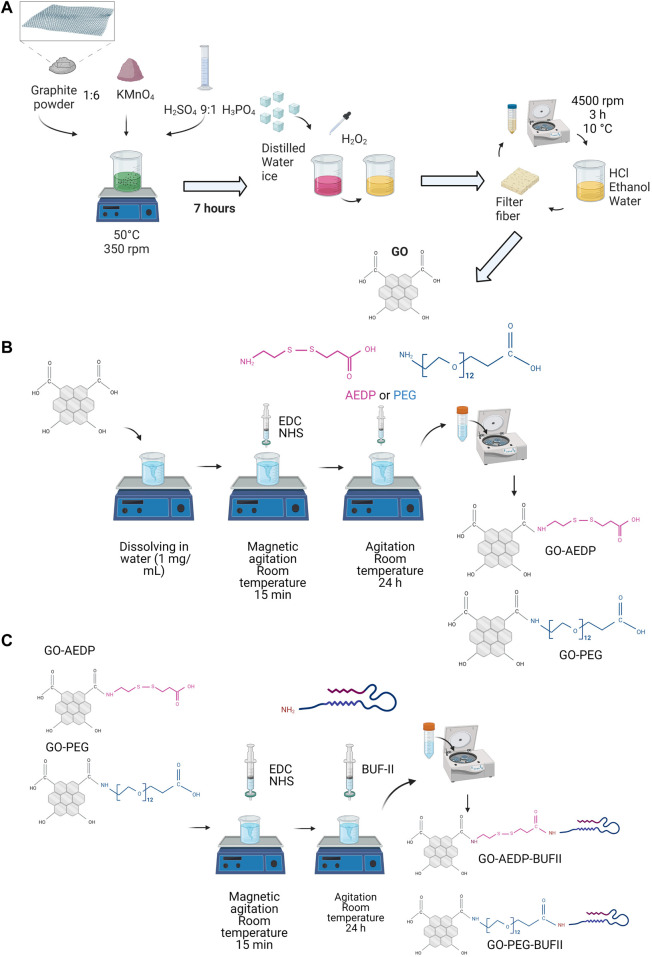
Scheme of the experimental procedure for constructing the nanobioconjugates. **(A)** Synthesis of GO; **(B)** Functionalization of GO with reducible and polymeric linkers; **(C)** Conjugation of BUFII. Figure created with BioRender.com.

To prepare the GO nanosheets, a modification of Hummer’s method was conducted (Jankovský et al., 2017). This method consists of two main steps: the oxidation of graphite to obtain graphite oxide and its subsequent acidic exfoliation to finally produce graphene oxide (GO) (Figure 1A). The oxidation process was conducted by mixing graphite powder (0.75 g) with potassium permanganate (4.5 g) and a liquid solution (100 ml) of sulfuric acid (90% v/v) and phosphoric acid (10% v/v). This reaction mixture was left under continuous magnetic agitation for 7 h at 50°C. Next, the mixture was rapidly cooled down to room temperature. Distilled water ice (150 ml) and hydrogen peroxide (2 ml) were added to the mix to quench the oxidation reaction. The resulting solution was filtered to remove excess reagents with the aid of a polyester fiber. The exfoliation started by centrifuging the solution at 4,000 rpm, 10°C (ThermoFisher Scientific Sorvall Legend XTR, Osterode, Germany). The resulting pellet was resuspended in an aqueous solution composed of HCl (50 ml, 37% v/v), ethanol (50 ml, 96%), and deionized water (50 ml). To ensure a homogeneous pellet dispersion in the acidic solution, samples were sonicated for 15 min in an ultrasonic bath (Bransonic 2510R-DTH, Danbury, CT, United States). This process was repeated five times to produce highly-dispersed GO nanosheets. After the exfoliation process, samples were lyophilized for 48 h and stored at 4°C for further characterization.

### 2.3 Functionalization of GO

GO nanosheets were functionalized in a two-step process to produce two nanobioconjugates using the EDC/NHS chemistry, which facilitates the formation of amide bonds. In the first step, a solution containing 10 mg of EDC and 20 mg of NHS was dripped in 40 ml of GO (2.5 mg/ml) at a rate of 1 ml/min under continuous magnetic stirring. After adding EDC and NHS, the solution was left under agitation for 15 min. Upon activating carboxyl groups via EDC/NHS, 5 mg of AEDP or PEG (NH_2_-PEG_12_-Propionic acid) molecules were added to the corresponding samples, followed by continuous agitation for 24 h. To remove the excess reagents, samples were centrifuged and resuspended in deionized water. After this, two nanoconjugates functionalized with either the AEDP (GO-AEDP) or PEG molecules (GO-PEG) were obtained (Figure 1B).

The antimicrobial and cell-penetrating peptide BUF-II was then immobilized by taking advantage of the terminal carboxyl groups of the reducible and polymeric linkers, AEDP, and PEG molecules. To do this, 10 mg of EDC and 20 mg of NHS were added to 40 ml of both nanoconjugates GO-AEDP and GO-PEG (Figure 1C). Samples were subsequently left under continuous stirring for 24 h. To remove the excess reagents from the functionalization process, samples were centrifuged and resuspended in type I water. The resulting nanobioconjugates (GO-AEDP-BUFII and GO-PEG-BUFII) were stored at 4°C for further characterization.

### 2.4 Characterization techniques

#### 2.4.1 Spectroscopic analysis

To corroborate the synthesis and functionalization processes for the obtention of GO-based nanobioconjugates, Fourier-Transform infrared spectroscopy (FTIR) analyses were performed. Small solid dry samples (approximately 5 mg) of bare GO and GO-based nanobioconjugates were analyzed via a Bruker Alpha II FTIR Eco-ATR instrument (Bruker, Billerica, MA, United States). The spectra were collected by averaging three scans taken at a resolution of 2 cm^−1^ in the spectral range of 4,000–400 cm^−1^.

To determine the extent of oxidation of bare GO and to confirm the two-step functionalization process, Raman spectroscopy analyses were also conducted. Solid dry samples were analyzed via an XploRA Confocal Raman Microscope (Horiba Scientific, Japan).

#### 2.4.2 Thermal stability analysis

Thermogravimetric analyses were performed to estimate the efficiency of the conjugation of BUF-II on the surface of GO. Three mg of solid dry samples were analyzed via a simultaneous TGA/DSC instrument (TA Instruments, New Castle, DE, United States). The analyses were conducted at a linear heating rate of 10°C/min from 25 to 600°C under a nitrogen atmosphere. The conjugation efficiency was estimated by correlating the percentage of weight loss to the detachment of the molecules chemically bound to the surface of GO.

#### 2.4.3 Microscopic analysis

To analyze the morphology of the nanosheets for bare and modified GO, the samples were imaged via scanning electron microscopy (SEM, TESCAN LYRA3 FIB-SEM, Brno, Czech Republic) and transmission electron microscopy (TEM, Tecnai F30 Microscope (Fei Company, Hillsboro, OR, United States).

In addition, height profiles of the nanosheets were obtained via atom force microscopy (AFM 3D MPF Bio, Asylum Research/Oxford Instruments, California, United States) to estimate their thicknesses. A sharp silicon AC24TS scanning probe (with a radius of 7 nm and an elastic constant of 1 nN/nm—according to the manufacturer) was used to register high-resolution topographical maps of the surface samples. To deposit the samples for this analysis, small silicon chips were cleaned with RCA1 (a solution containing NH4OH and H2O2) and RCA2 (a solution composed of HCl and H2O2) and dried with nitrogen (see details of the cleaning protocol in (Pillers et al., 2015)). After this cleaning process, each bare and modified GO liquid samples were deposited between two silicon chips like a “sandwich.” The samples were heated at 40°C to remove excess water.

### 2.5 Biocompatibility assays

#### 2.5.1 Hemolysis assay

To evaluate the hemolytic activity of the nanobioconjugates, erythrocytes isolated from the freshly drawn blood of a healthy human donor were exposed to the nanostructures. The blood sample was centrifuged at 1800 rpm for 5 min to obtain the erythrocytes, and the plasma (supernatant) was discarded. After the precipitation of the erythrocytes, they were washed thrice by cyclically centrifuging and resuspending them in PBS (1X). A stock containing 1 ml of the isolated erythrocytes and 9 ml of PBS (1X) was prepared upon washing the erythrocytes. The nanobioconjugates were serially diluted in PBS (1X) from 200 to 12.5 μg/ml. Triton X-100 (1% (v/v)) and PBS (1X) were selected as positive and negative controls, respectively. After serially diluting the nanobioconjugates, 100 μl of each treatment were seeded with 100 μl of the erythrocytes and incubated at 37°C, 5% CO_2_ for 1 h. After the incubation, samples were centrifuged at 1800 rpm for 5 min, and 100 μl of the supernatant were placed in a 96-well microplate to read their absorbance at 450 nm via a Multiskan FC microplate reader (Thermo Fisher Scientific, Waltham, MA, United States). Hemolysis percentage was calculated as indicated in Eq. (1):

$$\%\;Hemolysis=100∙\frac{\left(Absorbance\;of\;sample-Absorbance\;of\;negative\;control\right)}{\left(Absorbance\;of\;positive\;control-Absorbance\;of\;negative\;control\right)}\;$$ (1)

#### 2.5.2 Platelet aggregation assay

To determine the extent of platelet aggregation of the nanobioconjugates, platelets extracted from freshly drawn blood of a healthy human donor were exposed to the nanostructures. The blood sample was stored in a vacutainer tube with sodium citrate as an anticoagulant to isolate the platelets, avoiding aggregation. To obtain the platelet-rich plasma (PRP), the blood sample was centrifuged at 1,000 rpm for 15 min at room temperature. The nanostructures were serially diluted in PBS (1X) from 200 to 12.5 μg/ml. Thrombin was used as a positive control, while PBS (1X) worked as a negative reference. 50 μl of the PRP was pipetted into 50 μl of the nanobioconjugates in a 96-well microplate. After seeding the platelets, samples were incubated at 37°C, 5% CO_2_ for 5 min. Upon incubation, absorbance was read at 620 nm via a microplate reader. Platelet aggregation percentage was calculated by dividing the absorbance of the sample by the absorbance of the control.

#### 2.5.3 MTT cytotoxicity assay

The cytotoxicity of bare GO, GO-AEDP, GO-PEG, GO-AEDP-BUF-II, and GO-PEG-BUF-II was evaluated in Vero (ATCC®CCL-81) and THP-1 (ATCC^®^ TIB-202) cells ((American Type Culture Collection, Manassas, VA, United States). Vero cells are derived from the kidney of an African green monkey and are a model line close to fibroblasts, primarily present in human tissues due to their ability to form connective tissue. This makes it one of the more commonly used mammalian continuous cell lines in microbiology and molecular and cell biology research (Ammerman et al., 2008). THP-1 is a human leukemia monocytic cell line, which has been extensively used to study monocyte/macrophage functions, mechanisms, signaling pathways, and nutrient and drug transport (Chanput et al., 2014). To do the cytotoxicity tests, a colorimetric assay based on the metabolic conversion of 3-(4,5-dimethylthiazol-2-yl)-2,5-diphenyltetrazolium (MTT) into formazan by active cells was applied. The nanostructures were serially diluted from 200 to 12.5 μg/ml. Non-supplemented DMEM medium was used as the negative control. 100 µl of a cell suspension stock in DMEM medium supplemented with FBS (10%) was seeded in a 96-well microplate at 10 × 10^4^ cells/well. After this, the microplates were incubated at 37°C, 5% CO_2_, and in a humidified atmosphere for 24 h. Upon incubation, cells were exposed to the different treatments for 24 and 48 h by extracting and replacing a 10-percent-FBS-supplemented DMEM medium with a non-supplemented DMEM medium containing the different nanobioconjugates. After exposure, 10 µl of MTT reagent (5 mg/ml) was added to each well, and the microplates were then incubated as described above for 2 hours. After the action of the MTT reagent, supernatants were discarded, and 100 µl of DMSO was added to each well to dissolve the resulting formazan crystal. As the last step, a microplate reader (Thermo Scientific Multiskan™ FC Microplate Photometer) was used to record the absorbance at 595 nm. Cell viability was computed as the division between the absorbance of the sample and that of the negative control. GraphPad Prism V 8.0.1 software (GraphPad Software, La Jolla, CA, United States) was employed to compare the results statistically. The statistical comparisons were made through an unpaired *t*-test. Results with *p* < 0.05 (*) were considered significant.

### 2.6 Endosomal escape analysis

#### 2.6.1 Labeling of GO-based nanobioconjugates

To track the nanobioconjugates GO-AEDP-BUFII and GO-PEG-BUFII during the confocal microscopy analyses, these were labeled with the fluorophore Rhodamine B. This labeling was followed by the same EDC/NHS conjugation protocol used to functionalize GO. As a first step, 30 mg of EDC and 15 mg of NHS were chemically activated by dissolving them in a solution with 5 ml of type I water. After this, 2 ml of DMF and 5 mg of Rhodamine B were added to the solution and stirred for 15 min at 40°C under dark conditions to activate the carboxyl groups of the Rhodamine B and avoid photobleaching. Upon the chemical activation of Rhodamine B, the resulting solution was added to 100-mg samples of each nanobioconjugate. The final samples were stirred via a Compact Digital Mini Rotator shaker (Thermo Fisher Scientific, Seoul, Korea) at 200 rpm for 24 h. To remove excess reagents, the samples were thoroughly washed by centrifuging and resuspending them in Type I water. This washing process was cyclically repeated until a transparent supernatant of the samples was obtained. Finally, the samples were stored at 4°C under complete darkness until further use.

The nanomaterial GO, and the GO-AEDP and GO-PEG nanoconjugates served as controls. These were labeled following the same EDC/NHS conjugation protocol to track them during the confocal microscopy analyses. However, propidium iodide (5 mg) was used in this case as the fluorescent marker instead of Rhodamine B.

#### 2.6.2 Confocal microscopy analysis

The Rhodamine-B- and the Propidium-Iodide-labeled GO-based nanobioconjugates were delivered in Vero and THP-1 cells to analyze internalization. Briefly, one hundred thousand cells per well were seeded on a circular glass slide of 18 mm diameter previously coated with Poly-D-Lysine. Vero cells were incubated in DMEM medium supplemented with 5% (v/v) FBS for 24 h (37 C, 5% CO_2_) to allow cell adhesion, while THP-1 were maintained in RMPI medium supplemented with 10% (v/v) FBS (37°C, 5% CO_2_). After incubation, cells were exposed to the Rhodamine B-labeled nanobioconjugates for 0.5 and 4 h in a non-supplemented medium, at concentrations of 25 and 12.5 μg/ml for Vero and THP-1 cells, respectively. Cells were then exposed to a DMEM or RPMI solution with Hoechst 33,342 (1:10,000) and Lysotracker Green DND-26 (1:10,000 for 5 min to label nuclei and endosomes, respectively. Ten images of each treatment (10 cells per image) were then taken via an Olympus FV1000 confocal laser scanning microscope (CLSM) with a 40X/0.6 UCPlan FL N and a PlanApo 60X oil immersion objective. The nuclei, endosomes, and nanobioconjugates were detected at excitation/emission wavelengths of 405 nm/461 nm, 488 nm/535 nm, and 559 nm/600 nm, respectively. The surface area coverage by the nanobioconjugates and Pearson Correlation Coefficient (PCC) between the nanobioconjugates and Lysotracker Green^®^ were calculated to estimate the extent of internalization and endosomal escape of the nanobioconjugates. The Fiji-ImageJ^®^ software was used to analyze the collected images (Schindelin et al., 2012). GraphPad Prism V 8.0.1 software (GraphPad Software, La Jolla, CA, United States) was employed to statistically compare the results. The statistical comparisons were made through an unpaired *t*-test. Results with *p* < 0.05 (*) were considered significant.

## 3 Results

### 3.1 Characterization of nanobioconjugates

#### 3.1.1 Spectroscopic analysis

**FIGURE 2:**
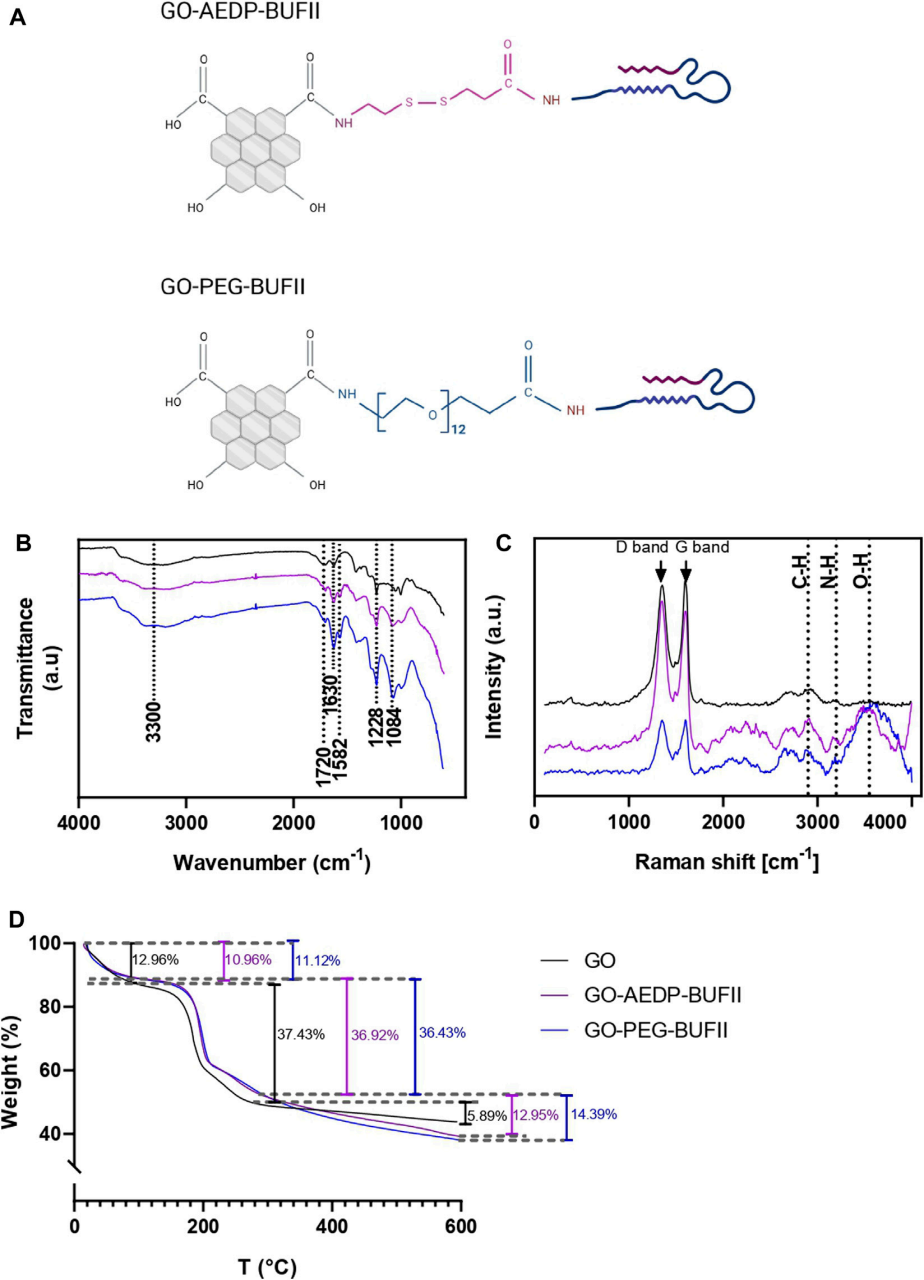
Characterization of the GO-AEDP-BUFII and GO-PEG-BUFII. **(A)** Schematic of the chemical structure of the developed nanobioconjugates. The obtention of these nanobioconjugates was successfully corroborated via: **(B)** FTIR spectral analyses; **(C)** Raman spectroscopic analyses; **(D)** TGA profiles.

Figure 2A shows a schematic of the final chemical structures of the GO-AEDP-BUF-II and GO-PEG-BUF-II nanobioconjugates. To confirm the correct synthesis of these nanobioconjugates, spectral analyses were conducted via FTIR (Figure 2B) and Raman (Figure 2C) spectroscopy.

The distinctive chemical groups of GO, GO-AEDP-BUF-II, and GO-PEG-BUF-II were identified by recording their characteristic infrared spectral bands (Figure 2B). For the GO, an ample peak at 3,300 cm^−1^ can be attributed to the stretching vibration of hydroxyl groups (O-H) (Marcano et al., 2010; Jankovský et al., 2017). Moreover, the carboxyl and epoxy groups were identified by the peaks at 1720 and 1,228 cm^−1^, attributed to the stretching vibrations of C=O and C-O bonds, respectively (Marcano et al., 2010; Jankovský et al., 2017). Additionally, a peak at 1,630 cm^−1^ was attributed to the stretching vibration of C=C bonds in the GO’s laminar structure (Marcano et al., 2010; Jankovský et al., 2017). For the GO-AEDP-BUFII and GO-PEG-BUFII nanobioconjugates, additional characteristic bonds were detected in their spectra, including the bending vibration of the N-H bond that was observed at 1,582 cm^−1^ (Perez et al., 2019), which was attributed to the amine groups of BUF-II. In addition, the amide bonds formed after functionalization, which was corroborated by the C-N stretching vibration observed at 1,084 cm^−1^ (Liu et al., 2016).

Additional information about the chemical structure of GO, GO-AEDP-BUF-II, and GO-PEG-BUF-II was obtained by collecting their Raman spectra (Figure 2C). GO-based nanostructures showed two major bands: The D-band (at 1,350 cm^−1^) and the G-band (at 1,600 cm^−1^). The D-band was attributed to defects in the laminar structure graphene layer. These defects can be explained by the sp^3^ hybridized carbon atoms, which indicate the presence of functional groups in the nanosheets (Marcano et al., 2010; Jankovský et al., 2017). In contrast, the G-band reflects the presence of sp^2^-bonded carbon atoms in the graphene layer without defects (Marcano et al., 2010; Jankovský et al., 2017). After calculating the ratio of the intensity of these two bands (i.e., I_D_/I_G_) for GO, GO-AEDP-BUF-II, and GO-PEG-BUF-II, we obtained 0.96, 1.07, and 0.99, respectively. There was a slight increase in such ratio GO-based nanobioconjugates, which was expected because the D-band reflects the presence of amine, hydroxyl, and ketone groups of the peptide structure.

#### 3.1.2 Thermal Stability analysis

The thermal stability of GO, GO-AEDP-BUF-II, and GO-PEG-BUF-II was determined via TGA analyses. These results are shown in Figure 2D, where three significant weight losses are noticeable: The first one below 100°C can be attributed to the evaporation of residual water from the samples. The second one (between 100 and 200°C) was related to the degradation of oxygen-containing chemical functionalities on the surface of the graphene layer. Furthermore, the third one above 300°C can be explained by the degradation of the graphene laminar structure. Both GO-based nanobioconjugates showed a larger total weight loss than the unmodified GO. The efficiency of the two-step conjugation process for GO-AEDP-BUF-II and GO-PEG-BUF-II was estimated at 7 and 8%, respectively.

#### 3.1.3 Microscopic analysis

**FIGURE 3:**
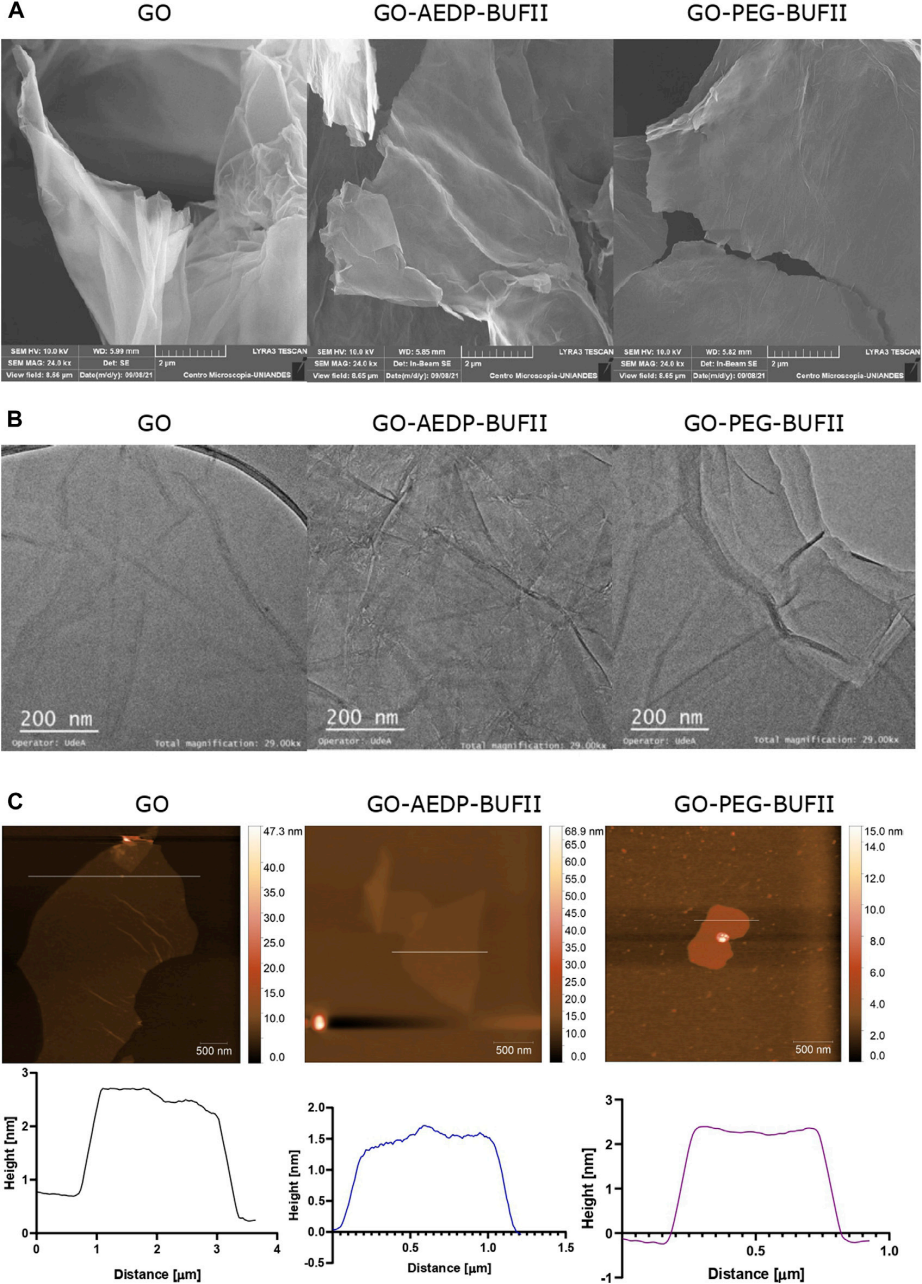
Microscopic characterization of the developed nanostructures via **(A)** SEM imaging; **(B)** TEM imaging; **(C)** AFM imaging, and height profiles.

The morphology of GO, GO-AEDP-BUF-II, and GO-PEG-BUF-II was analyzed via SEM and TEM images, shown in Figures 3A,B, respectively. Figure 3A shows that bare GO nanosheets exhibited marked wrinkles while GO-AEDP-BUFII and GO-PEG-BUF-II looked smoother. GO-PEG-BUFII nanosheets appeared smoother than GO-AEDP-BUF-II, which may be attributed to the larger polymeric linker, PEG_12_, compared with AEDP. This size difference in each linker may play a significant role in promoting steric hindrance that prevents nanosheet folding. This agrees well with the higher peaks observed in the Raman spectra (Figure 2C) of GO-PEG-BUFII compared to GO-AEDP-BUFII. Similar results were obtained via TEM images (Figure 3B) for nanosheets smaller than 200 nm.

To determine the thickness of GO, GO-AEDP-BUFII, and GO-PEG-BUFII nanosheets, height profiles were obtained from AFM images (Figure 3C). As shown in the height profiles of Figure 3C, the maximum thickness for GO, GO-AEDP-BUFII, and GO-PEG-BUFII rounded about 2.2, 1.7, and 2.7 nm, respectively.

### 3.2 Biocompatibility

**FIGURE 4:**
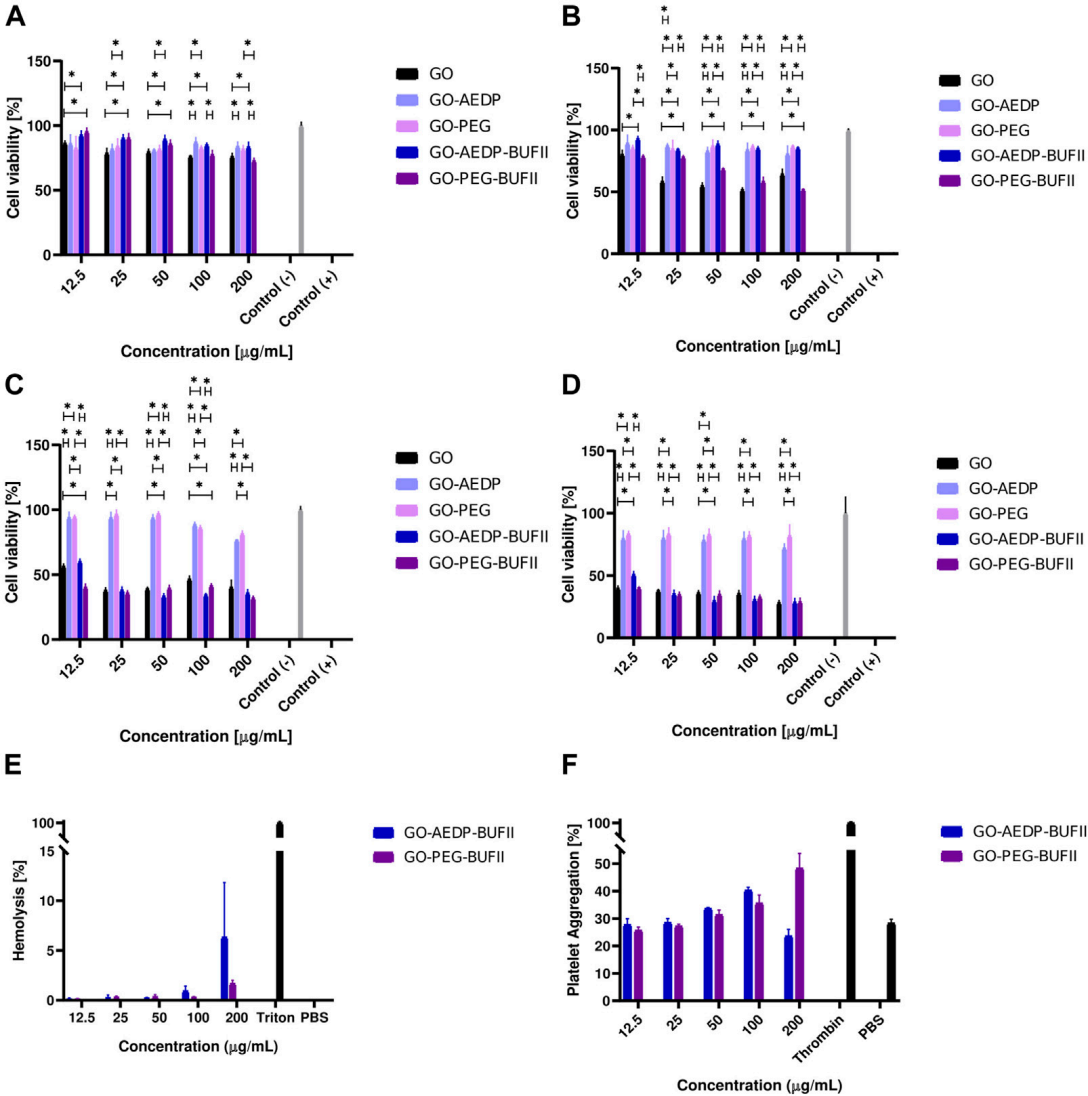
Biocompatibility tendencies of the developed nanobioconjugates. Cytotoxicity tendencies were evaluated in: Vero cells after incubation with treatments for **(A)** 24 h, **(B)** 48 h; and THP-1 cells after incubation with treatments for **(C)** 24 h, **(D)** 48 h. Results marked with (*) indicate significant differences between them (*p*-value<0.05). Hemocompatibility assays were conducted to determine: **(E)** Hemolytic effect and **(F)** Platelet aggregation levels.

Figures 4A,B show the cell viability after exposure of Vero cells to the nanomaterial GO and the GO-AEDP and GO-PEG nanoconjugates tructures for 24 and 48 h, respectively. After 24 h of exposure to the treatments up to 200 μg/ml, all nanostructures showed cell viability levels above 80%. However, this viability level significantly decreased to about 60% after 48 h of exposure to GO and GO-PEG-BUF-II, while cells exposed to GO-AEDP-BUF-II maintained their cell viability level. The modification of GO with AEDP and PEG significantly reduces the viability of Vero Cells after 24 h of exposure at concentrations above 100 μg/ml and after 48 h at concentrations above 25 μg/ml (Figures 4A,B). Furthermore, the subsequent immobilization of the peptide BUFII significantly reduces cell viability in Vero cells after exposure for 48 h (Figure 4B). In contrast to the excellent cytocompatibility observed in Vero cells, THP-1 cells were considerably affected by the exposure to GO and the GO-AEDP-BUFII and GO-PEG-BUFII nanobioconjugates (Figures 4C,D). After exposure for 24 and 48 h, the cell viability levels were reduced to about 40% in this cell line. However, the nanoconjugates without BUFII showed cell viability levels above 80%in Vero cells.

Regarding the hemocompatibility of our nanobioconjugates, Figure 4E shows that the hemolytic level of the nanobioconjugates remained below 10% for concentrations up to 200 μg/ml. Interestingly, GO-AEDP-BUF-II showed a considerably higher hemolytic effect than GO-PEG-BUF-II at 200 μg/ml. Figure 4F shows that the platelet aggregation effect of the nanobioconjugates remained below 48% for concentrations up to 200 μg/ml. For concentrations below 100 μg/ml, the aggregation level approached that of the negative reference, PBS 1X.

### 3.3 Internalization and endosomal escape

Visual indication of the nanobioconjugates GO-AEDP-BUFII and GO-PEG-BUFII’s ability to penetrate Vero and THP-1 cells is shown in Supplementary Figures S1 and S2. Although both nanostructures exhibited an excellent ability to enter both cell lines, cell morphology appears significantly altered for THP-1 compared with Vero. Supplementary Figures S3, S4, and S5 show internalization of GO, GO-AEDP, and GO-PEG in Vero and THP-1 cells.

**FIGURE 5:**
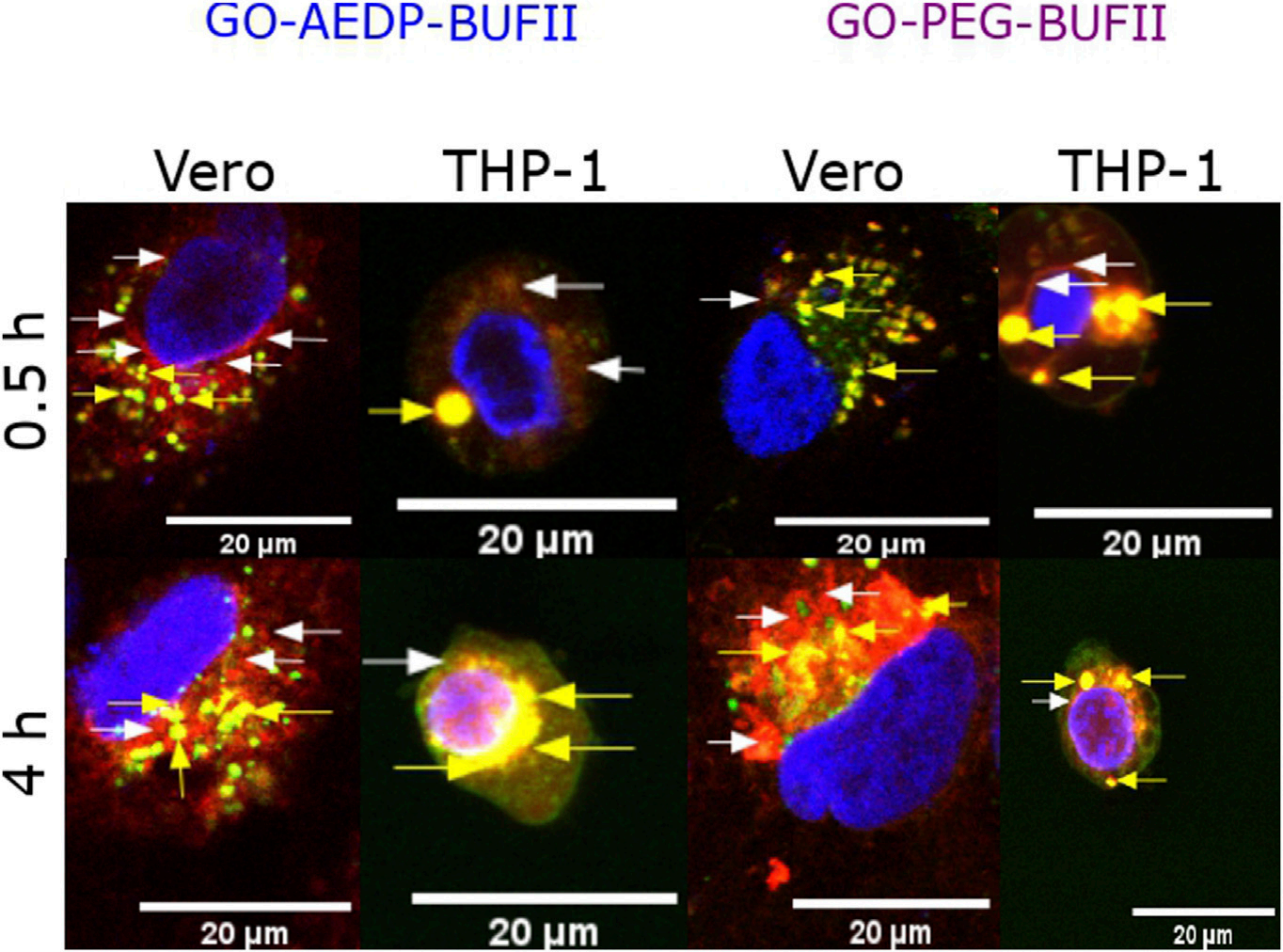
Visual inspection of colocalization studies via confocal imaging. Yellow arrows show the colocalization between the nanobioconjugates (red channel) and endosomes (green channel). White arrows show the absence of colocalization (nanostructures escaping from endosomes).

To determine the endosomal escape ability of the nanobioconjugates labeled with Rhodamine B, confocal microscopy analyses were conducted to look for colocalization of the nanobioconjugates with Lysotracker Green^®^. Figure 5 illustrates visual indications of the ability of the nanostructures to escape from endosomes. This ability was quantitatively estimated by calculating the Pearson correlation coefficient (PCC). This coefficient varies from 0 (indicating complete endosomal escape) to 1 (suggesting an absence of any endosomal escape).

**FIGURE 6:**
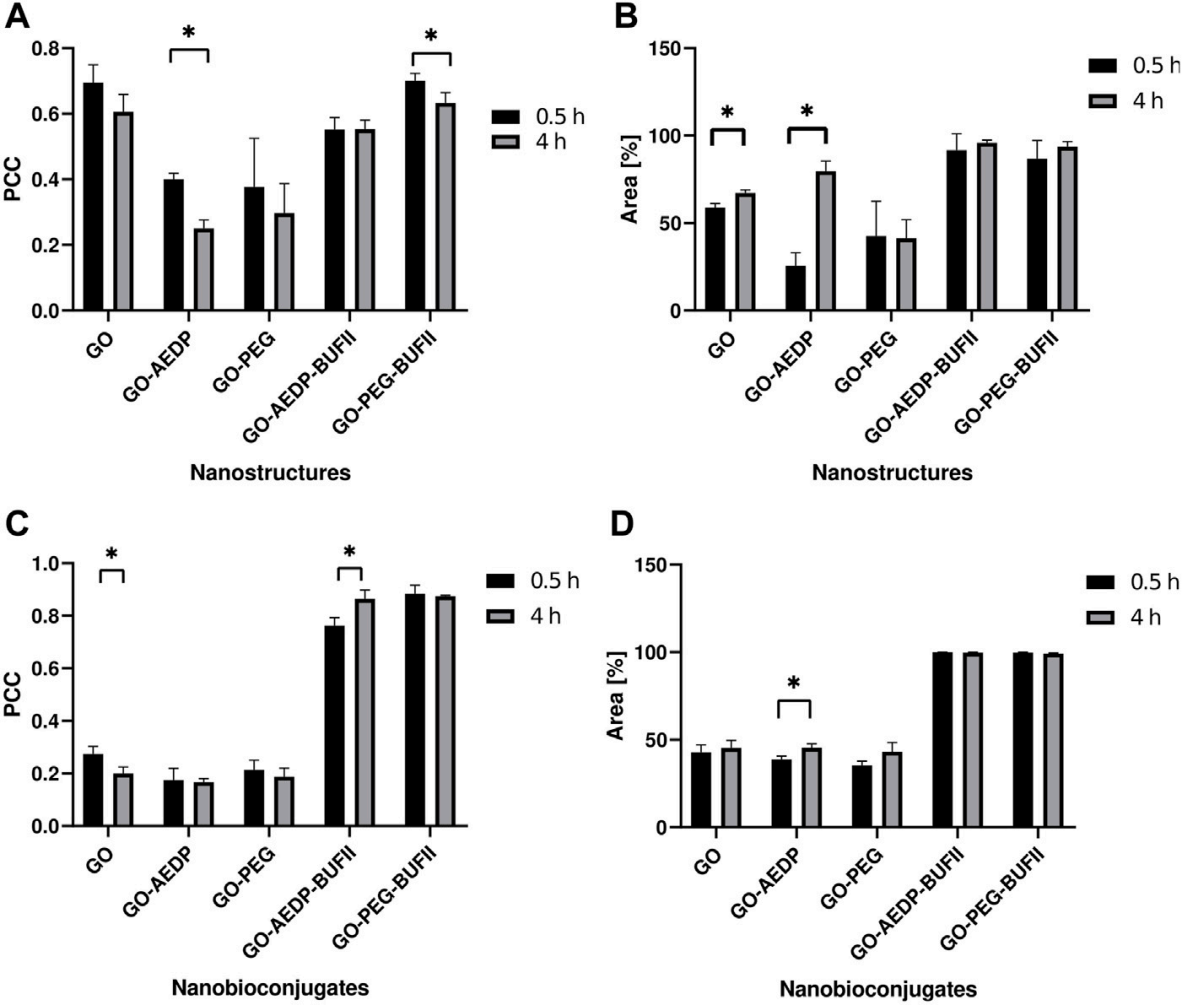
Statistical analysis of the confocal images. Endosomal escape and internalization of the nanobioconjugates were quantitatively analyzed by evaluating Vero cells to observe tendencies in: **(A)** Colocalization with endosomes by calculating the PCC values; **(B)** Covered area of the nanobioconjugates. Similarly, THP-1 cells were analyzed to look for tendencies in **(C)** Colocalization with endosomes; **(D)** Coverage area percentage of the developed nanostructures. Results marked with (*) indicate significant differences between them (*p*-value<0.05).

Results of the PCC of the nanobioconjugates internalized in Vero cells are shown in Figure 6A. The PCC of GO-AEDP-BUFII was estimated at 0.550 ± 0.001 after incubation for 0.5 h with no additional changes after 4 h. This value was the lowest of all evaluated treatments, indicating the best endosomal escape abilities among the nanobioconjugates tested in Vero cells. The GO-PEG-BUFII showed a PPC of 0.701 ± 0.022 after incubation for 0.5 h and a further decrease to 0.633 ± 0.031 after incubation for 4 h. The PCC decreased over time for bare GO and nanoconjugates in the absence of immobilized BUF-II. However, this reduction was statistically significant only for GO-AEDP, which showed a PCC of 0.401 ± 0.018 after incubation for 0.5 h and a further reduction to 0.250 ± 0.026 after 4 h.

To obtain a quantitative measure of how well our nanoconjugates enter the cells, the cell coverage area percentage was estimated using the Fiji ImageJ^®^ software. Results regarding cell coverage of the nanobioconjugates upon internalization in Vero cells are shown in Figure 6B. Cells exposed to GO-AEDP-BUFII for 0.5 h showed an area coverage percentage of 91,676 ± 9,348%. After 4h of exposure, there was an increase to 95.923 ± 1,482%, which was not statistically significant. A similar tendency was observed for GO-PEG-BUFII, which reached a coverage in Vero Cells of 86.840 ± 10,315 and 93.654 ± 2.895 after incubation for 0.5 and 4 h. Interestingly, bare GO only could reach an area coverage percentage of 58.842 ± 2.378% after incubation for 0.5 h, and significantly increased to 67.171 ± 1.820% after incubation for 4 h. Moreover, GO-AEDP showed an area coverage percentage of 25.542 ± 7.565% after incubation for 0.5 h, followed by a significant increase to 79.639 ± 5.822% after 4 h. GO-PEG showed a non-significant reduction in the area coverage percentage level over time. After 0.5 h of incubation, this value reached 42.587 ± 19.904% and slightly decreased to 41.371 ± 10.606% after 4 h of exposure.

Figure 6C shows the colocalization analyses for the nanobioconjugates in THP-1 cells. In contrast to the superior endosomal escape abilities observed in Vero cells, GO-AEDP-BUFII showed a tendency to be progressively captured by endosomes. This was evidenced by the PCC, estimated at 0.763 ± 0.029 and 0.864 ± 0.034 after incubation for 0.5 and 4 h, respectively. This suggests that the nanobioconjugates can internalize THP-1 cells through the interplay of non-endocytic pathwaysConversely, GO-PEG-BUFII showed a low endosomal escape ability in THP-1 cells. This can be corroborated by the slightly decreasing tendency of PCC values, estimated at 0.883 ± 0.033 and 0.874 ± 0.004 after incubation for 0.5 and 4 h, respectively. However, this reduction was not statistically significant. For the nanoconjugates with no immobilized peptide, the PCC was below 0.3. Over time, the nanostructures failed to show a significant difference in the PCC, except for GO, which showed a PCC of 0.275 ± 0.028 and 0.200 ± 0.025. In terms of cell internalization in THP-1 cells, both GO-AEDP-BUFII and GO-PEG-BUFII showed an almost complete internalization in THP-1 cells, as shown in Figure 6D. Oppositely, nanostructures lacking the peptide only could reach an area coverage percentage close to 50%.

## 4 Discussion

### 4.1 Synthesis and characterization of nanobioconjugates

Previous contributions in our research group have been dedicated to describing various magnetite-based nanoplatforms formed by interfacing magnetite nanoparticles with translocating proteins and peptides (Cuellar et al., 2018; Perez et al., 2019; Ramírez-Acosta et al., 2020). This study expands the use of cell-penetrating peptides to the graphene-based nanomaterials, which were obtained by functionalizing GO with reducible molecules (to favor delivery of cargoes under typical cytosolic redox conditions) and polymeric spacers (to facilitate cell internalization and maintain high cytocompatibility) onto which the translocating peptides were conjugated. Our work, therefore, features a robust methodology for the synthesis and functionalization of GO to obtain novel carbon-based nanoplatforms with cell-penetrating and endosomal escape abilities.

GO was functionalized to obtain two different nanoplatforms: the first one by functionalizing it with a reducible linker (AEDP), followed by conjugation of BUFII antimicrobial peptide Buforin II, while for the second one, we employed a heterobifunctional carboxyl-amine-ended-polymeric molecule (PEG) to which BUFII was conjugated. Both nanoplatforms were successfully characterized via spectral analyses, which confirmed the presence of the expected chemical bonds, including hydroxyls, carboxyls, and epoxies for GO (Marcano et al., 2010; Jankovský et al., 2017). Additionally, the presence of amine groups for the GO-AEDP-BUFII and GO-PEG-BUFII nanobioconjugates (Perez et al., 2019) and the amide bonds formed after the functionalization processes (Liu et al., 2016). Thermogravimetric analyses demonstrated proper immobilization of BUFII with an efficiency close to 7% (for GO-AEDP-BUFII) and 8% (for GO-PEG-BUFII). These values are close to those determined in previous works for the conjugation of BUFII to magnetite nanoparticles (Cuellar et al., 2018; Perez et al., 2019; Ramírez-Acosta et al., 2020). The morphology of the nanobioconjugates was effectively determined via microscopic analyses, showing smooth nanosheets as previously reported by authors following similar functionalization approaches to develop drug delivery systems (Chen et al., 2021; Mirza-Aghayan et al., 2022). The thicknesses reported in this study (less than 3 nm) are in the range of previous reports for graphene layers (Chen et al., 2021; Mirza-Aghayan et al., 2022; Molaparast et al., 2022).

### 4.2 Biocompatibility

The development of novel nanovehicles for medical purposes requires evaluating possible biocompatibility issues to elucidate whether these nanovehicles can be further considered for pre-clinical and clinical assays. In this regard, we conducted cytotoxicity, hemolytic, and platelet activity assays with the synthesized nanobioconjugates. Figures 4A,B show that Vero cells showed viability levels up to 80% after 24 h of exposure to the nanobioconjugates. Interestingly, the modification of GO with AEDP or PEG does not considerably compromise the viability of Vero cells. However, after immobilizing BUFII on the GO-PEG’s surface, the cell viability level in this cell line was reduced by 60% after 48 h of exposure. This might indicate that the peptide immobilized in a conformation that favor its interaction with the cell membrane and other cellular components, most likely altering intracellular trafficking and eventually leading to a viability reduction. This behavior might be due to the larger size of the PEG spacer compared to AEDP.

In contrast to this tendency, cells maintained viability levels above 80% after 48 h of exposure to the GO-AEDP-BUFII nanobioconjugates. This might indicate that incorporating a reducible linker may not significantly reduce viability for Vero cells. Previous reports have corroborated enhanced nanocarrier biocompatibility upon immobilizing reducible molecules (Li et al., 2014). This was not the case for THP-1 cells, which were considerably affected by the exposure to bare GO and the GO-AEDP-BUFII and GO-PEG-BUFII nanobioconjugates (Figures 4C,D). After exposure for 24 and 48 h, the cell viability levels were reduced to about 40%, which has been previously reported for GO in THP-1 cells (Gurunathan et al., 2019). However, GO-based nanoconjugates without the peptide showed cell viability levels above 80%. This suggests that the peptide remained bioactive after immobilization on GO, and its interaction with the cellular membrane and eventual intracellular trafficking might alter the metabolism of THP-1 cells, thereby leading to death. This opens a need to study further the possible cell membrane interactions and intracellular trafficking pathways involved upon cellular internalization and to search for novel approaches to further engineer our nanobioconjugates to maintain their potent cell-penetrating abilities without compromising the viability of THP-1 cells.

Nanomaterial-based cell-penetrating vehicles are primarily administered intravenously. For this reason, evaluating the hemolytic and platelet aggregation activities of our nanobioconjugates is critical to determine whether they can be suitable for such an administration route. As shown in Figure 4E, the hemolytic level of the nanobioconjugates remained below 10% for concentrations up to 200 μg/ml. Interestingly, GO-AEDP-BUFII showed a higher hemolytic level compared with GO-PEG-BUFII at 200 μg/ml. The use of reducible molecules and PEG-based polymers to covalently functionalize GO has been reported previously as a strategy to enhance hemocompatibility (Yang et al., 2010; Yang et al., 2012b; de Melo-Diogo et al., 2018). In parallel, the platelet aggregation level of the nanobioconjugates remained below 48% for concentrations up to 200 μg/ml (Figure 4F). For concentrations below 100 μg/ml, the aggregation level approached that of the negative reference, PBS 1X. Taken together, these biocompatibility findings indicate that the developed nanobioconjugates are suitable for their intended application and encourage us to advance to pre-clinical studies.

### 4.3 Internalization and endosomal escape of nanobioconjugates

In terms of cell internalization, our nanobioconjugates tend to rapidly penetrate cells compared to other nanovehicles, in which this process can last for up to 8 h (Schweiger et al., 2012). This is confirmed by the high cell coverage area levels of the nanostructures, which ranged from 76 to 100% for Vero cells (Figure 6B) and approached 100% for THP-1 cells (Figure 6D). Conversely, those nanoconjugates lacking BUF-II showed cell coverage area levels ranging from 40 to 70%. This highlights the crucial role of BUF-II in the cellular internalization of our nanobioconjugates. These findings align with our previous studies on developing cell-penetrating magnetite nanobioconjugates based on BUFII (Perez et al., 2019; Ramírez-Acosta et al., 2020). This ability might be associated with the synergy between the antimicrobial peptide BUFII and the PEG spacer. This cationic peptide can induce the translocation of cell membranes due to its interaction with negatively charged components of the bilayer through the transient formation of peptide-lipid supramolecular complexes of toroidal shape that might act as pores (Kobayashi et al., 2004; Koren and Torchilin, 2012; Cuellar et al., 2018; Ramírez-Acosta et al., 2020). This capacity has been already demonstrated by us for magnetite-BUF-II nanobioconjugates (Cuellar et al., 2018; Perez et al., 2019; Ramírez-Acosta et al., 2020). Another critical factor is the surface chemistry obtained after the functionalization process. Figure 6B shows that the conjugation of a reducible spacer in GO’s surface leads to a significant increase in cell coverage area percentage over time. Previous studies have shown that molecules containing a disulfide bond in their structure may be reduced during the interaction with cellular membranes, leading to translocation processes (Gasparini et al., 2014).

The endosomal entrapment of nanomaterial-based cell-penetrating systems (e.g., the nanobioconjugates) is a critical drawback for the efficient delivery of different therapeutic cargoes (Selby et al., 2017; Donahue et al., 2019). This entrapment has been thought to be related to a multi-pathway endocytic mechanism that involves either clathrin-mediated or caveolin-mediated endocytosis (Bareford and Swaan, 2007; Hillaireau and Couvreur, 2009; Ayala et al., 2013; Parton and Del Pozo, 2013; Mayor et al., 2014; Elkin et al., 2016; Donahue et al., 2019). While our previous studies showed potent endosomal escape abilities approaching levels ranging from 60 to 85% (Perez et al., 2019; Ramírez-Acosta et al., 2020), our GO-based nanobioconjugates reached endosomal escape levels ranging from 12 to 45% (Figures 6A,C). These differences might be associated with possible interactions of bare GO’s chemical moieties (e.g., epoxies, hydroxyls, and carboxyls) with the lipidic bilayer of the endosomal membrane (Feng et al., 2021). However, further studies need to be conducted to uncover the impact of the core nanomaterial on the ability of the nanobioconjugates to escape from endosomes. Another factor to consider is the impact of the resulting surface chemistry after completion of the cell-penetrating nanobioconjugate’s synthesis. This could be observed in the significant reduction of the PCC over time for GO-AEDP in Vero cells. Based on previous work, we hypothesize that the disulfide bond reduction might trigger endosomal membrane disruption due to interactions with the resulting thiol group (Gasparini et al., 2014).

Our results indicate that incorporating a reducible molecule into the nanobioconjugates’ structure confers a rapid endosomal escape ability in Vero cells. This might be attributed to the release of BUF-II upon reaching the cytosol, which triggers an endosomal breakdown. It has been hypothesized that the disruption of the endosomal membrane may be mediated by either a proton sponge effect (Yezhelyev et al., 2008; Freeman, Weiland and Meng, 2013; Thapa and Sullivan, 2018; Lopez-Barbosa et al., 2020; Ramírez-Acosta et al., 2020) or by interactions between the membrane and the peptide, triggering the formation of pores causing the eventual endosomal escape (Kobayashi et al., 2004). The use of reducible molecules has been previously reported in similar systems to facilitate the release of cargoes under physiological reducing environments, such as those found in the cytoplasm (Kam, Liu and Dai, 2005; Park et al., 2013). In parallel, polymeric spacers facilitate cellular uptake and a progressive endosomal escape afterward. The PEGylation of nanomaterials has been widely used for the development of nanovehicles because they confer more stability, cytocompatibility, and immunological properties to the PEGylated nanostructures upon cellular uptake (Oishi et al., 2006; Yang K. et al., 2012; Shen et al., 2012; Feng et al., 2014; Sun et al., 2016; Lopez-Barbosa et al., 2020). Although intracellular entrapment in endosomes/lysosomes is the preferred destination of nanoplatforms internalized in cells, a small fraction has been reported to reach the nuclear region (Figure 5). Our research group previously reported this capability for the magnetite-BUF-II nanobioconjugates (Cuellar et al., 2018; Perez et al., 2019; Ramírez-Acosta et al., 2020). This opens an avenue for the development of graphene-based gene delivery systems. However, details about the ability to penetrate the nuclear membrane must be explored further in future work. Unlike what was observed in Vero cells, our nanobioconjugates were mainly captured by endosomes in THP-1 cells. Only 12–15% of the nanostructures escape from endosomes in these cells. For some of the developed nanobioconjugates, the endosomal entrapment increased over time. This might indicate their internalization via the interplay of multiple pathways (e.g., caveolin-mediated and/or clathrin-dependent endocytosis, membrane translocation) (Hillaireau and Couvreur, 2009). Therefore, future studies will be focused on elucidating the relative contribution of such pathways and the possible intracellular trafficking routes and underlying mechanisms.

Furthermore, the endosomal capture of the nanostructures in THP-1 cells might be attributed to the previously reported toxic dose-dependent dependence of GO in such cells. This effect has been attributed to the stimulatory cellular mechanisms such as ROS proliferation and the imbalance of antioxidants, which may lead to the activation of pro-apoptotic gene expression cascades and the concomitant suppression of anti-apoptotic gene expression. As a result, the secretion of various cytokines and chemokines is notably affected (Gurunathan et al., 2019). Additionally, this is consistent with the cytotoxicity trends observed in this work. These results reflect the impact of different functionalization strategies on the ability of graphene-based nanoplatforms to penetrate various cell lines and escape from endosomes upon cellular uptake. However, the specific interactions triggering a different stimulation of these cytotoxic mechanisms in each cell line is still an underlying factor requiring further studies.

In summary, the cell-penetrating abilities of our nanobioconjugates were verified. The developed nanostructures took less than 30 min to completely cover the cell cytoplasm in both cell lines, while the absence of the cell-penetrating peptide led to a significantly reduced cell coverage area in both cell lines. This reflects a rapid cell internalization compared to most reported cell-penetrating nanoplatforms. In terms of endosomal escape properties, different behaviors were observed depending on the chemical structure of the nanobioconjugate and the cell line. The cleavable linkage in the GO-AEDP-BUFII nanobioconjugates showed a rapid endosomal escape process in Vero cells. This was reflected in a Pearson Correlation Coefficient (PCC) of about 55%, the lowest value reported in this study. We hypothesized that this might be associated with the breakdown of the disulfide bond under the reducing conditions of the cytosol.

In contrast, nanostructures with a polymeric linker showed a slow but progressive endosomal escape process. We believe that the presence of PEG-based molecules confers chemical stability, cytocompatibility, and immunological properties to the nanobioconjugates that enable steady intracellular trafficking, which agrees well with several similar reports of carbon-based delivery systems. Unlike the tendencies observed in Vero cells, the nanobioconjugates were strongly entrapped by endosomes in THP-1 cells. This was reflected in a PCC in the 76–86% range. Regarding these findings, this study shows the impact of changing the functionalization strategy on the cell-penetrating and endosomal escape processes taking place in different cell lines. Further studies become vital to explore more details about the pathways involved in these cellular processes. Considering that endosomal escape is a critical drawback for the delivery of therapeutic agents, this study opens an avenue to engineer functionalization strategies to develop novel, effective graphene-based delivery systems.

## Data Availability

The raw data supporting the conclusions of this article will be made available by the authors, without undue reservation.
