# Supplementary material for: Assessing cellular internalization and endosomal escape abilities of novel BUFII-Graphene oxide nanobioconjugates
Source: Front Chem. 2022 Sep 15;10:974218. doi: 10.3389/fchem.2022.974218 (PMC9521742; doi:10.3389/fchem.2022.974218)
Supplement: Supplementary file 1 [file DataSheet1.docx]

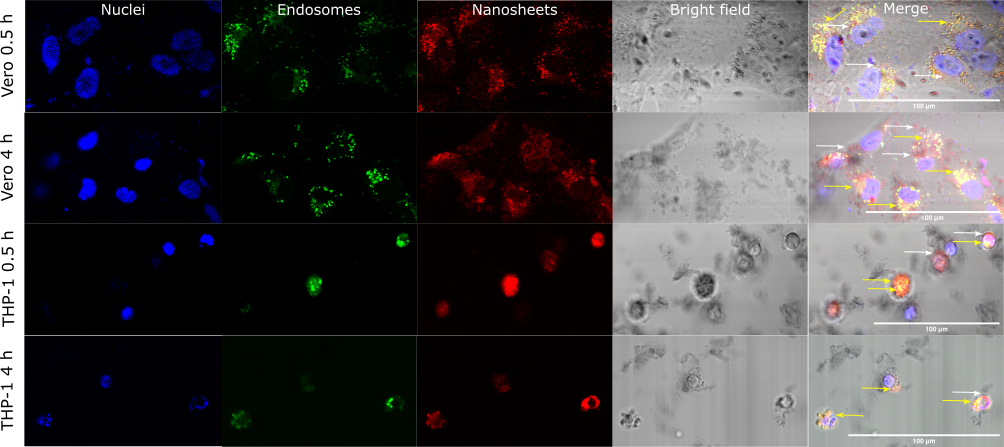


**Figure S1.** Confocal images of Vero and THP-1 cells after incubation with GO-AEDP-BUFII nanobioconjugates for 0.5 and 4 hours. Yellow arrows point to regions of low colocalization between the nanobioconjugates (red channel) and endosomes (green channel). White arrows indicate regions of low colocalization (i.e., nanobioconjugates escaping from endosomes).

**
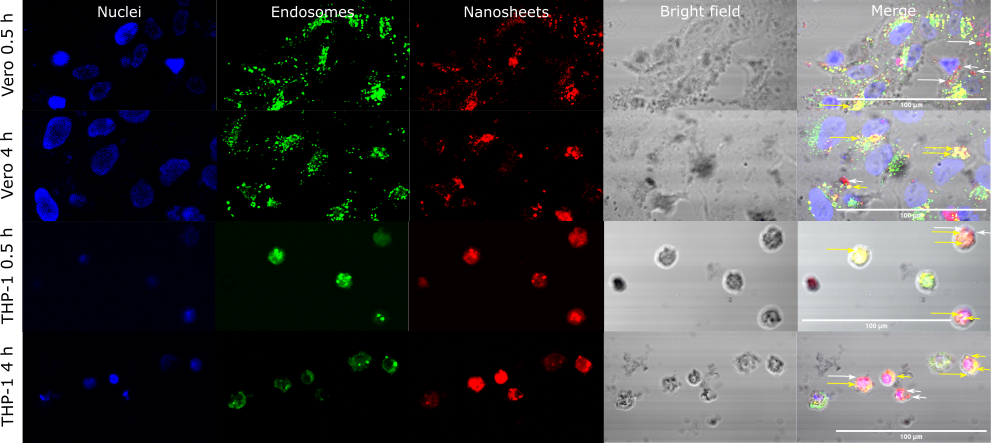
**

**Figure S2.** Confocal images of Vero and THP-1 cells after incubation with GO-PEG-BUFII nanobioconjugates for 0.5 and 4 hours. Yellow arrows point to regions of high colocalization between the nanobioconjugates (red channel) and endosomes (green channel). White arrows indicate regions of low colocalization (i.e., nanobioconjugates escaping from endosomes).


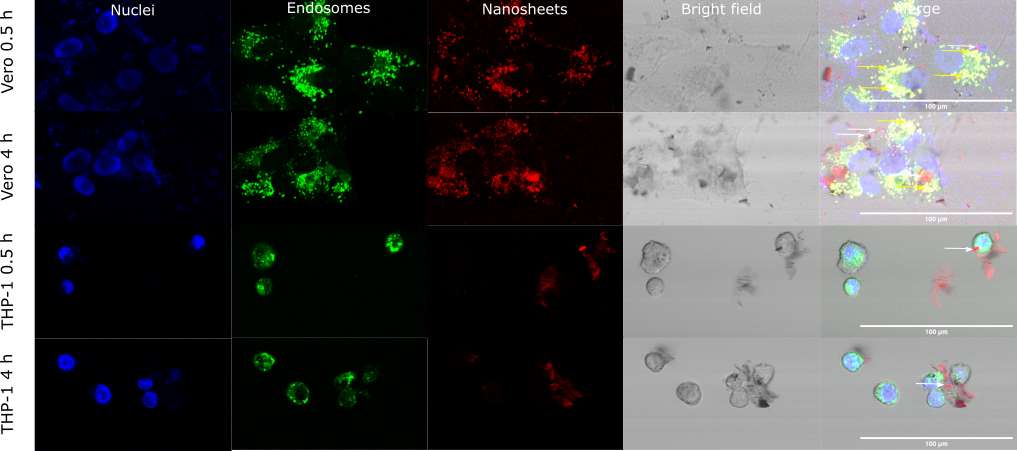


**Figure S3.** Confocal images of Vero and THP-1 cells after incubation with GO nanobioconjugates for 0.5 and 4 hours. Yellow arrows point to regions of high colocalization between the nanobioconjugates (red channel) and endosomes (green channel). White arrows indicate regions of low colocalization (i.e., nanobioconjugates escaping from endosomes).


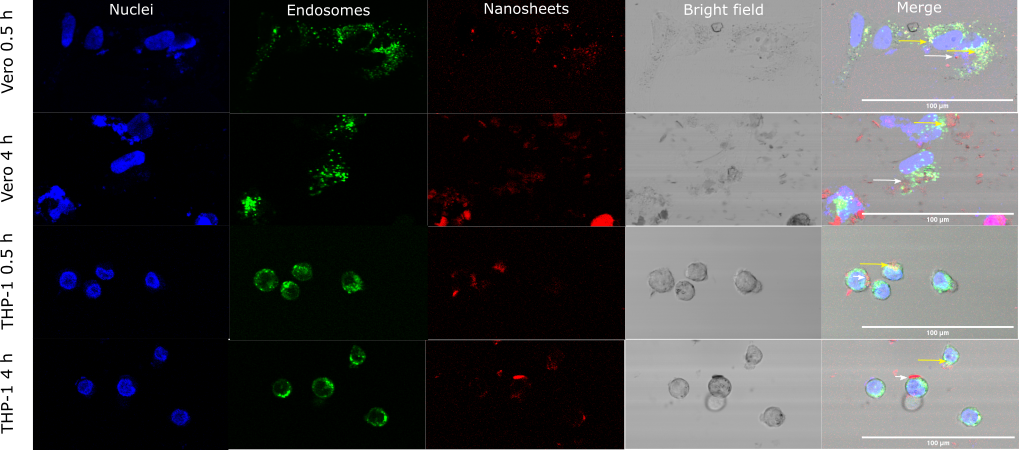


**Figure S4.** Confocal images of Vero and THP-1 cells after incubation with GO-AEDP nanobioconjugates for 0.5 and 4 hours. Yellow arrows point to regions of high colocalization between the nanobioconjugates (red channel) and endosomes (green channel). White arrows indicate regions of low colocalization (i.e., nanobioconjugates escaping from endosomes).


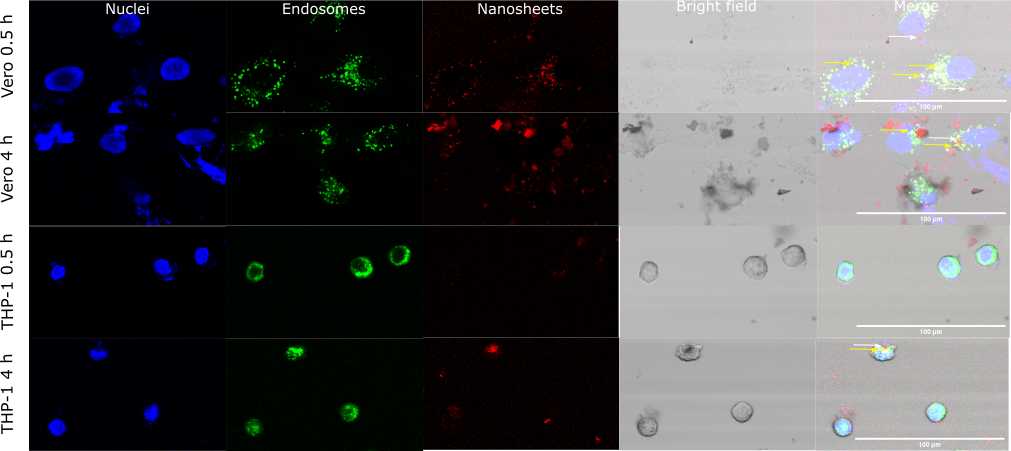


**Figure S5.** Confocal images of Vero and THP-1 cells after incubation with GO-PEG nanobioconjugates for 0.5 and 4 hours. Yellow arrows point to regions of high colocalization between the nanobioconjugates (red channel) and endosomes (green channel). White arrows indicate regions of low colocalization (i.e., nanobioconjugates escaping from endosomes).
